# Intrahousehold empowerment gaps and dietary diversity in China

**DOI:** 10.3389/fnut.2024.1365652

**Published:** 2024-03-20

**Authors:** Yanfang Huang, Yuying Yang, Fengying Nie, Xiangping Jia

**Affiliations:** ^1^Institute of Agricultural Information, Chinese Academy of Agricultural Sciences, Beijing, China; ^2^School of Agricultural Economics and Rural Development, Renmin University of China, Beijing, China

**Keywords:** intrahousehold empowerment gaps, women's empowerment, gender inequality, dietary diversity, China

## Abstract

**Objective:**

This article analyzes the relationship between intrahousehold empowerment gaps and food and nutrition security using quantitative data collected through a household survey organized by the Agricultural Information Institute, Chinese Academy of Agricultural Sciences (CAAS-AII), in 2023.

**Methods:**

Based on empowerment theory, this study measured the relative empowerment of spouses from the Abbreviated Women's Empowerment in Agriculture Index (A-WEAI).

**Results:**

From the micro-level evidence of 468 rural households, this study found that intrahousehold empowerment gaps harm the diversity of household diets. In particular, reducing gender gaps in access to resources, leadership, and income can help diversify household diets. However, data on the impact of shortening the difference in working hours between wives and husbands for the benefit of food safety are yet to be conclusive. Additionally, gender gaps in the group of non-coresident mothers-in-law and non-migrants hurt household food security.

**Conclusion:**

The paper also provides further justification for policies and interventions that aim to improve women's bargaining position in the household.

## 1 Introduction

The number of challenges to eliminating hunger, food insecurity, and all forms of malnutrition keep rising (1). In 2020, the average global cost of a healthy diet was USD 3.54 per person per day, reflecting a 3.3 and 6.7% increase from 2019 and 2017, respectively. The number of people unable to afford a healthy diet globally has increased by 112 million to nearly 3.1 billion due to increased food prices from 2019 to 2020 (1), which further increases world hunger, severe food insecurity, and different forms of malnutrition.

However, current policies supporting food and agriculture need to reduce hunger, food insecurity, and malnutrition more effectively (1). It has been widely recognized that low levels of dietary diversity are unfavorable for the transition of agrifood systems to higher nutritional values (2, 3). Increasing women's empowerment is essential for wellbeing and positively impacts food security, diet diversity, and child nutrition (1, 4–16).

Recently, the global development discourse has recently shifted from food security to food and nutrition security, emphasizing the need to understand gender dynamics in agriculture to attain global nutritional goals (17–21). Specifically, several empirical studies in development economics have attempted to identify the potential benefits of expanding women's agency in the household. Historically, the simplest family decision-making models have depended on a unitary household model (22, 23). Such models effectively suppose that family members pool resources and share the same preferences. Yet, a considerable body of research suggests that the relative decision-making abilities of both husbands and wives within the household are closely related to the allocation of resources in the collective family (e.g., income, food, health care, etc.); in other words, most households do not necessarily pool their resources, and spousal preferences can be viewed as heterogeneous in many contexts (24–31). Thus, household welfare outcomes are affected by the relative decision-making ability of males and females within the same household (9, 24, 25, 32–34).

The Yunnan province of China is a region primarily populated by ethnic minorities characterized by widespread low levels of education and poor diet quality. Gender norms deeply permeate the power dynamics within families, and both spouses are influenced by traditional gender culture (25, 35). Only the husband's preferences determine the distribution of resources and income, and women's family status is low. However, to our knowledge, the gender gaps in decision-making are rarely integrated when studying dietary diversity and directing policy interventions in transforming the agrifood system toward a healthy diet (36, 37).

This paper draws from the theory of “intra-household bargaining” offered by Sen and illustrates how inequality between different household members affects decision-making processes and the allocation of resources (38). The results of this study are consistent with a range of developing county contexts (39–42), emphasizing the shifts in women's decision-making abilities, which have led to changes in welfare and other outcomes for women and households.

Our study aims to bring fresh insights into the relationship between gender inequality and food consumption within agricultural households in China. First, we broadly measured empowerment levels based on the empowerment theory (43) and the developed tools of the Abbreviated Women's Empowerment in Agriculture Index (A–WEAI) (6, 44, 45), considering matters beyond single- or few-household decisions (46) that may have missed meaningful variation in spousal ability across different decisions. Second, unlike prior studies (47), this paper reports on both spouses' relative authority, which is essential to understanding how intrahousehold empowerment gaps affect household development.

## 2 Methods and materials

### 2.1 Study area

**Figure 1:**
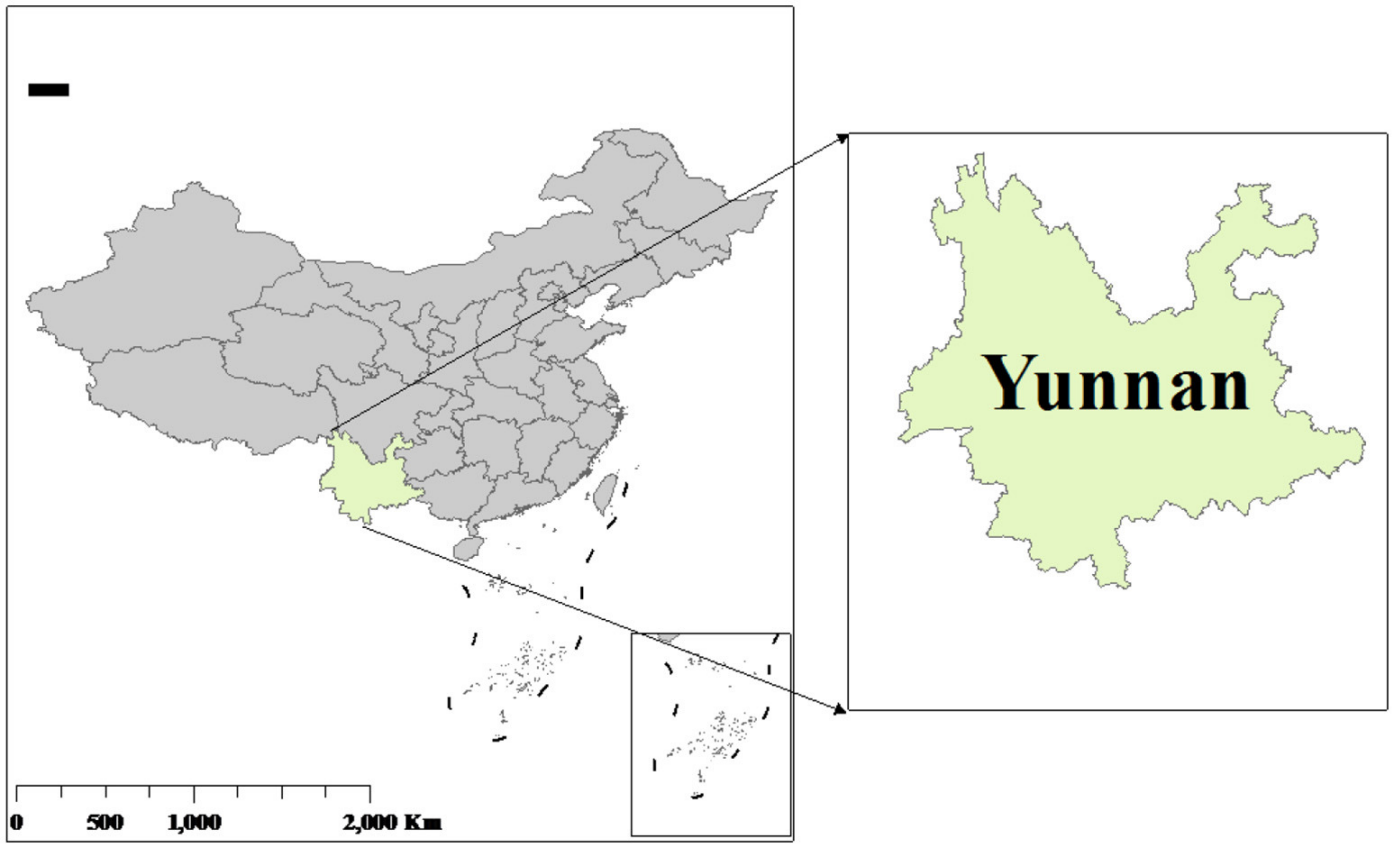
Map of the study area.

The study was conducted in Jiangcheng County and Simao district of Yunnan Province, located in Southwestern China (see Figure 1). Mountains characterize the landscape of these areas, and the average altitude is above 1,300 meters. Agricultural production is mainly cultivated through drought-tolerant crops (such as corn) and cash crops (flue-cured tobacco and tea); 98.76% of the cultivated land is not irrigated, and irrigation depends on the weather (48). In 2023, the households' per capita net income amounted to CNY 20,661, with 55% attributed to agricultural production. A monotonous diet remains a significant problem for smallholder farmers in these districts, as they mainly consume grains and vegetables, and consuming eggs, milk, and meat is insufficient (36). Women are primarily responsible for tea picking in these areas, spending an average of 7.56 hours per day engaged in this activity^1^. In addition, women are responsible for caring for the family, devoting ~3 h per day to this activity, much more than those of men (49).

### 2.2 Study design and sampling

We employed quantitative data from a household survey facilitated by the Agricultural Information Institute of the Chinese Academy of Agricultural Sciences (CAAS-AII). Random sampling was used for our research including counties (*N* = 2) covered by the CAAS-AII program, town (*N* = 6), village (*N* = 13), and households (*N* = 520).

The survey targeted currently married male and female households. A combined total of 500 women (96.2% response rate) and 513 men (98.7% response rate) were successfully interviewed. As the primary interest of the present study was intrahousehold gender gaps and food consumption, we restricted our sample to 489 married houeholds, in whom both wife and husband were interviewed. Further excluding couples with missing data in certain items, our final sample for this study consisted of 468 households (Table 1).

**Table 1:** Sample description of the study.

**County**	**Town (N)**	**Village (N)**	**Household (N)**
Jiangcheng	3	7	284
Simao	3	6	184
Total	6	13	468

### 2.3 Data collection and variables

Data collection was conducted between March and April 2023 and was completed by seven researchers and 30 enumerators. Before the formal research, we tested the questionnaire content in non-sample households (*N* = 111) and finalized the questionnaire after several rounds of revision and discussion with enumerators and researchers.

As the primary interest of the present study was intrahousehold gender gaps and food consumption, the research team behind this paper developed specific modules on the topics at the individual, gender and household levels. Specifically, individual modules include the general demographic characteristics of household members; gender-specific modules, e.g., male and female personal social network, mental health status, attitudes toward risk and value, time and social preferences, and perceptions of empowerment; household modules contain general information on the household's agricultural production, financial situation, food consumption, income and expenditure, and risk shocks. Among them, the male answered all questions in the survey, and the female answered the same questions in gender-specific modules. Spouses were interviewed separately and individually to reduce response bias.

The outcome variable of interest in this study was the household dietary diversity. Dietary data was collected on consumption status in twelve food groups of the household in the past day, including cereals, stems, vegetables, fruits, meat, eggs, fish and seafood, milk, legumes, fats, sugars, and condiments. To reduce recall bias, enumerators surveyed households about their food consumption the previous day on the following morning by asking, “Did you eat this food yesterday?”.

Eventually, this paper following the FAO's “food group-based indicators” (FGIs) method–household dietary diversity score (HDDS)–to measure household dietary diversity (50, 51). The households' 24-h food consumption recall data was categorized into twelve major food groups, each food group was assigned one point based on whether they had been consumed in the past day and vice versa (52, 53).

The key explanatory variable was the empowerment gaps, measured using the Relative Empowerment Score (RES). Mainly, we collected empowerment data for wives and husbands separately and individually by asking, “If the respondent participates in an agricultural income-generating activity individually and jointly,” “If the respondent solely or jointly owns land, buildings, or vehicles,” “If the respondent participates in decision making concerning credit individually or jointly,” “If the respondent participates in decisions regarding the use of income,” “If the respondent is a member of at least one economic or social group,” and “If the respondent works more than 10.5 h a day, including household chores, care of children and elders, agricultural activities, non-farm production, etc.”. When the interviewee answers each question, a dummy variable is generated accordingly, i.e., 1 = yes; 0 = no. Noteworthy, assigned a value of 0 when respondent answered that he/she works more than 10.5 h a day, and vice versa to 1.

Then, based on the empowerment theory (43), we calculated the empowerment score by employing the developed tools of the Abbreviated Women's Empowerment in Agriculture Index (A–WEAI) (6, 44, 45). First, we calculated individual-level (male and female) empowerment adequacy scores for six indicators under five dimensions: production, resources, income, leadership, and time (Table 2). Next, weighted scores defined by the A–WEAI scoring scheme (45) were summed up to calculate the aggregated overall empowerment scores underlying the individual-level data. Lastly, we constructed a continuous relative empowerment score for each household by comparing the primary male and female respondents.

**Table 2:** Indicators for calculating the empowerment score.

**Domain**	**Indicator**	**Definition of adequacy**	**Weight**
Production	Input in productive decisions	If the respondent participates in an agricultural income-generating activity individually and jointly: 1 = Yes; 0 = No	1/5
Resources	Ownership of assets	If the respondent solely or jointly owns land, buildings, or vehicles: 1 = Yes; 0 = No	2/15
	Decisions on credit	If the respondent participates in decision making concerning credit individually or jointly: 1 = Yes; 0 = No	1/15
Income	Control over use of income	If the respondent participates in decisions regarding the use of income: 1 = Yes; 0 = No	1/5
Leadership	Group membership	If the respondent is a member of at least one economic or social group: 1 = Yes; 0 = No	1/5
Time	Workload	If the respondent works more than 10.5 h a day: 1 = No; 0 = Yes	1/5

Other covariables potentially associated with the dependent variable were selected based on the extant literature (36, 44), including the age of the household head, the education level of the household head, the health status of the household head, household size, the area of cultivated land, communication expenditure, household income, household wealth, social network, etc.

Information on the age of the household head (years), and the education level of the household head (years of formal education) from the husband's response. The household wealth variable measured by a five-item categorical variable, with five indicating very rich and one indicating extreme poor. The social network variable is also a five-item categorical variable; higher numbers represent a higher frequency of communication with other people. The health status variable is a binary variable; a value of one was assigned when the household head received medical treatment in the last 12 months. Otherwise, a value of zero was assigned. The household income variable was constructed by adding income from different income-generating activities (e.g., agricultural, non-agricultural, or both). The communication expenditure variable was created by summing the household members' cash expenditure on communication-related products, services, and activities (54, 55).

### 2.4 Data analyses

All data analyses were performed using the statistical software STATA v15 (56). Descriptive statistics were sourced from the mean and standard deviation of individual data for males and females, as well as other data related to the household level. A correlation test analyzed the associations between dietary quality and demographics. For dietary diversity, the HDDS values were divided into three groups following the Food and Nutrition Technical Assistance (FANTA) proposal (57): Low Diet Diversity (LDD) score ≤ six food groups; Medium Diet Diversity (MDD) score ≤ eight food groups; High Diet Diversity (HDD) score > eight food groups.

Multivariable regression models (using a *p*-value < 0.10 to define significance) were conducted to capture the correlation between each explanatory variable and outcomes regarding diet diversity, and the overall empowerment gaps were expanded into five specific dimensions (production, resources, income, leadership, and time). Furthermore, we used the household dietary diversity score based on nine major food groups (HDDS9)— cereals, stems, vegetables, fruits, meat, eggs, fish and seafood, milk, and legumes—as an alternative measure of diet diversity to examine the robustness of the results.

More importantly, Ordinary Least Squares (OLS) is used because the explanatory variables are uncorrelated with the disturbance term. However, the explanatory variables are often correlated with the disturbance term in reality, leading to inconsistency in the OLS. Therefore, we also estimated the correlation through the standard Instrumental Variable (IV) method (24, 46), and the validity of the instrumental variable was tested using a two-stage least squares (2SLS) method (58, 59). Specially, following Sraboni et al., we used an instrument variable, i.e., whether an area has suffered natural disasters during the previous year that are likely to be correlated with women's abilities to exercise agency and negotiate with their husbands and exogenous to the current period's decisions regarding household diet (44). Multicollinearity was evaluated using the variance inflation coefficient (VIF < 10) to remove any outliers from the analyses (60).

### 2.5 Empirical specification and estimating methods

When estimating the impacts of the empowerment gap on household dietary diversity in China, we specified the basic model as follows:


(1) $$\begin{array}{l} HDDS=\lambda_{0}+\lambda_{1}RES+\lambda_{2}\;HCG+\lambda_{3}H+\lambda_{4}\;I+\vartheta \end{array}$$

In Equation 1, *HDDS*, a continuous variable measuring dietary diversity at the household level, spans from zero to twelve, corresponding to the number of consumed food groups. *RES* presents the difference in male and female empowerment scores. Higher numbers indicate more significant empowerment gaps between husband and wife, and zero indicates perfect equality; the Human Capital Gap (*HCG*) measures the difference in male and female human capital for a household; it is a continuous variable; *H* and *I* represent household and individual characteristics, respectively, including the age of the household head, the education level of the household head, the health status of the household head, household size, the area of cultivated land, communication expenditure, household income, household wealth, social network (36, 44); λ_*i*_ are the parameters to be estimated; and ϑ is an error item.

## 3 Results

### 3.1 Descriptive statistics

Our study was conducted in areas that are considered targets for China's national food security measures. As shown in Table 3, both spouses are seemingly of a moderate age; the average age of the husband and wife was 51.2 and 49.5 years, respectively, with 23.4% of males and 19.0% of females aged 60 and above. The intermediate education level of both spouses was low (< 7 years), and the education level of males was 1 year higher than that of females. The diet diversity level of households in the sample area was low, with 63.3% of households below the average dietary score of 7.05. Regarding household wealth, 39.3% of households were poor or severely poor. In terms of household income, the per capita net income of the households was CNY 8,657.8 in 2023; according to China's 2022 poverty line standard (4,000 CNY/year), 23.9% of the region's population was below the poverty line.

**Table 3:** The baseline characteristics of all interviewers by gender.

**Variable**	**Total sample (*N* = 468)^a^**	**Male (*n* = 468)^b^**	**Female (*n* = 468)^b^**	**VIF**
**Individual characteristics**				
Age (years, mean ± SD)	51.00 ± 10.88^c^	51.15 ± 10.95	49.46 ± 11.78	1.33
Education (years, mean ± SD)	6.41 ± 3.28^d^	6.48 ± 3.22	5.47 ± 3.67	1.38
Health status (%)^e^				1.10
0 = no	60.04	–	–	–
1 = yes	39.96	–	–	–
Empowerment score (mean ± SD)	–	0.740 ± 0.209	0.670 ± 0.260	1.05
Production (%)^f^	–	86.32	73.50	–
Resources (%)^f^	–	89.96	69.02	–
Income (%)^f^	–	76.28	77.78	–
Leadership (%)^f^	–	10.90	12.83	–
Workload (%)^f^	–	36.54	35.47	–
**Household characteristics**				
HDDS (mean ± SD)	7.05 ± 1.49	–	–	–
Household size (person, mean ± SD)	4.11 ± 1.47	–	–	1.25
Household wealth (%)				1.10
1 = very poor	6.24	–	–	–
2 = poor	33.12	–	–	–
3 = average	58.49	–	–	–
4 = rich	1.94	–	–	–
5 = very rich	0.22	–	–	–
Area of arable land (mu, mean ± SD)	19.46 ± 15.27	–	–	1.11
Communication expenditure (CNY 1,000, mean ± SD)	2.37 ± 1.52	–	–	1.26
Household income (CNY 1,000, mean ± SD)	19.16 ± 36.09	–	–	1.24
Social networks (%)				1.03
1 = never communicate	12.23	–	–	–
2 = occasionally communicate	15.45	–	–	–
3=sometimes communicates	31.76	–	–	–
4=more often communicates	26.39	–	–	–
5 = often	14.16	–	–	–

Continuous variables are presented as Mean ± SD; Categorical variables are presented as %.

^a^Unit of *N* is the household.

^b^Unit of *n* is the individual.

^c^Denotes the age of the household head.

^d^Denotes the education level of the household head.

^e^Denotes the health status of the household head.

^f^Reference group: 0 = no.

Overall, the empowerment level of the wife was lower than that of the husband within the same household. The average empowerment score of males was 0.05 points higher than that of females. Specifically regarding the five dimensions of empowerment, men's empowerment in production, resources, and income exceeded women's by 12.8, 20.9, and 1.5%, respectively. Yet, men are less involved in the community than women (1.9%), and the number of men with a heavier workload was one percent higher than that of women.

### 3.2 Correlation analysis

**Figure 2:**
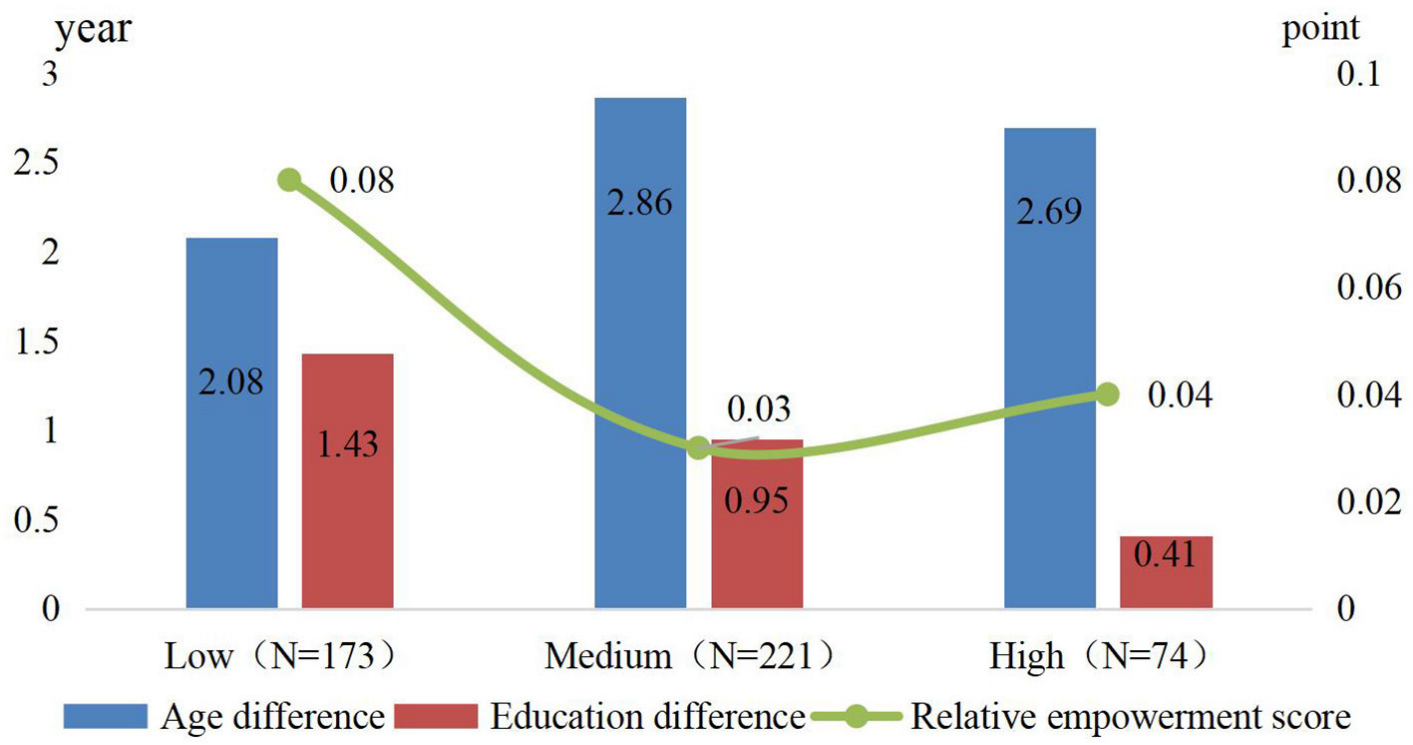
Spousal differences based on different household diet diversity scores (HDDS).

The diet diversity of the studied households correlates with the demographics. As shown in Figure 2, 84.2% of the households had a lower dietary diversity score than the target value (9.43) needed to assess household diet security. The age difference in the LDD group was 2.08 years, significantly lower than that of the HDD group. Similarly, the education difference decreased from 1.43 years in the LDD group to 0.41 years in the HDD group. This suggests that differences in capital can affect diet quality, even when other factors remain unchanged—the more significant the age difference between spouses, the higher the household's diet diversity level. Conversely, the narrower the gap in education levels, the more diverse the diet. In addition, the statistical results also showed that the relative empowerment score was negatively correlated with household diet diversity; the smaller the gender empowerment gaps, the more diverse the diet. Specifically, the LDD group had an empowerment gap of 0.08 points, significantly higher than the other two groups.

### 3.3 Multivariable regression models

Table 4 displays that intrahousehold empowerment gaps are detrimental to household diet diversity. The coefficient of the variable of RES was negative at a 1% level of significance (β = −0.56), indicating the negative impact of empowerment gaps on diet diversity. In particular, a unit increase in relative empowerment score decreased the likelihood of household diet diversity by 56% at a 1% significance level. This implies that households that close gender gaps are more likely to improve their diet quality.

**Table 4:** Multiple regression analysis identifying the influence of the empowerment gaps on household dietary diversity score (HDDS).

**Variables**	**Column 1**	**Column 2**	**Column 3**	**Column 4**
Relative empowerment score	−0.383^*^ (0.208)	−0.377^*^ (0.228)	−0.497^**^ (0.222)	−0.563^***^ (0.216)
Age difference		0.003 (0.012)	0.004 (0.011)	0.003 (0.010)
Education difference		−0.036^*^ (0.021)	−0.046^**^ (0.022)	−0.035 (0.022)
Age of household head			−0.006 (0.007)	−0.001 (0.007)
Education of household head			0.057^**^ (0.025)	0.045^*^ (0.025)
Health status of household head			−0.473^***^ (0.148)	−0.307^**^ (0.149)
Household size				−0.061 (0.057)
Household wealth				0.248^**^ (0.116)
Area of arable land				0.010^**^ (0.005)
Communication expenditure				0.110^**^ (0.051)
Household income				0.044 (0.089)
Social networks				0.113^*^ (0.059)
Constant	7.077^***^ (0.069)	7.163^***^ (0.083)	7.313^***^ (0.481)	6.118^***^ (1.016)
*N*	468	468	468	468

^*^p < 0.1, ^**^p < 0.05, ^***^p < 0.01.

The results in the third column show that the coefficient of education difference was negative and significant at a 5% significance level (β = −0.05), demonstrating that a unit percent increase in education difference resulted in a 0.05 point decrease in diet diversity score. This suggests a negative association between differences in capital and household diet diversity. However, when the household characteristic variables were incorporated into the model, the coefficient of education difference was non-significant, which means that human capital difference has less of an impact on household food security than household resources do.

Table 4 shows the diverse impacts of control variables on household dietary diversity. The coefficient for the education levels of household heads yielded a significant positive impact (β = 0.05), reflecting the beneficial impact of educational attainment. Household wealth conveyed that a one-unit increase led to a 0.25-unit rise in dietary diversity, highlighting the positive impact of improved wealth status. Similarly, land size demonstrated that a 1% increase resulted in a similar increase in dietary diversity. Communication expenditure indicated a significantly positive impact, implying a 25% rise in diet diversity with a 1% increase in expenditure. Significantly, social networks also had a positive impact, facilitating diet diversification. On the other hand, the household head's health status hinted at a substantial decrease in dietary diversity with a 1% deterioration. In contrast, other variables like the household head's age, household size, and income had no significant effect on household diet diversity in the model.

When examining the diverse effects of different empowerment domains on household diet diversity, we uncovered strong evidence suggesting that empowerment gaps diminish household food security. As presented in Table 5, we estimated other model regressions run separately on the five A-WEAI domains (expressed as dummy variables, where 1 indicates an empowerment gap, and 0 indicates no empowerment gap) with the same household and individual controls as Table 4.

**Table 5:** Male–female differences in five empowerment domains and household dietary diversity score (HDDS).

	**Production**	**Resource**	**Income**	**Leadership**	**Time**
Empowerment gaps	−0.174 (0.143)	−0.446^***^ (0.141)	−0.262^*^ (0.142)	−0.542^*^ (0.022)	0.237 (0.207)
Age difference	0.001 (0.010)	0.001 (0.010)	0.004 (0.010)	0.002 (0.010)	0.002 (0.010)
Education difference	−0.036 (0.022)	−0.035 (0.022)	−0.033 (0.022)	−0.037^*^ (0.022)	−0.038^*^ (0.022)
Age of household head	−0.002 (0.007)	−0.001 (0.007)	−0.003 (0.007)	−0.003 (0.007)	−0.002 (0.007)
Education of household head	0.042^*^ (0.025)	0.042^*^ (0.024)	0.041^*^ (0.025)	0.038 (0.025)	0.045^*^ (0.025)
Health status of household head	−0.270^*^ (0.149)	−0.287^*^ (0.148)	−0.253^*^ (0.149)	−0.241 (0.149)	−0.279^*^ (0.149)
Household size	−0.065 (0.057)	−0.060 (0.057)	−0.062 (0.057)	−0.065 (0.057)	−0.071 (0.057)
Household wealth	0.234^**^ (0.116)	0.234^**^ (0.115)	0.240^**^ (0.116)	0.224^*^ (0.116)	0.237^**^ (0.116)
Area of arable land	0.008 (0.005)	0.009^*^ (0.005)	0.009^*^ (0.005)	0.008 (0.005)	0.008^*^ (0.005)
Communication expenditure	0.112^**^ (0.051)	0.111^**^ (0.051)	0.116^**^ (0.051)	0.116^**^ (0.051)	0.113^**^ (0.051)
Household income	0.056 (0.089)	0.068 (0.088)	0.045 (0.089)	0.045 (0.089)	0.052 (0.089)
Social network	0.105^*^ (0.060)	0.102^*^ (0.059)	0.101^*^ (0.060)	0.116^*^ (0.060)	0.110^*^ (0.060)
Constant	6.135^***^ (1.023)	6.030^***^ (1.012)	6.281^***^ (1.024)	6.791^***^ (1.091)	5.913^***^ (1.040)
*N*	468	468	468	468	468

^*^p < 0.1, ^**^p < 0.05, ^***^p < 0.01.

The coefficient of empowerment gaps in the resource domain was negative and significant at a 1% significance level (β = −0.446). This implies that reducing the gender gaps in resource access can help diversify household diets. The estimates of empowerment gaps in leadership and income domains were also negative and significantly correlated with household diet diversity. This suggests that an increase in female's participation in groups and discretion over income relative to males is likely to improve diet quality. However, the coefficient of the empowerment gaps in the time domain was positive and had no significant effect on household diet diversity, indicating that reducing the difference in working hours between wife and husband is beneficial to family food safety under certain conditions. The coefficient of the education difference in leadership and time domain suggests a strong and negative association between human capital difference and household diet diversity.

**Figure 3:**
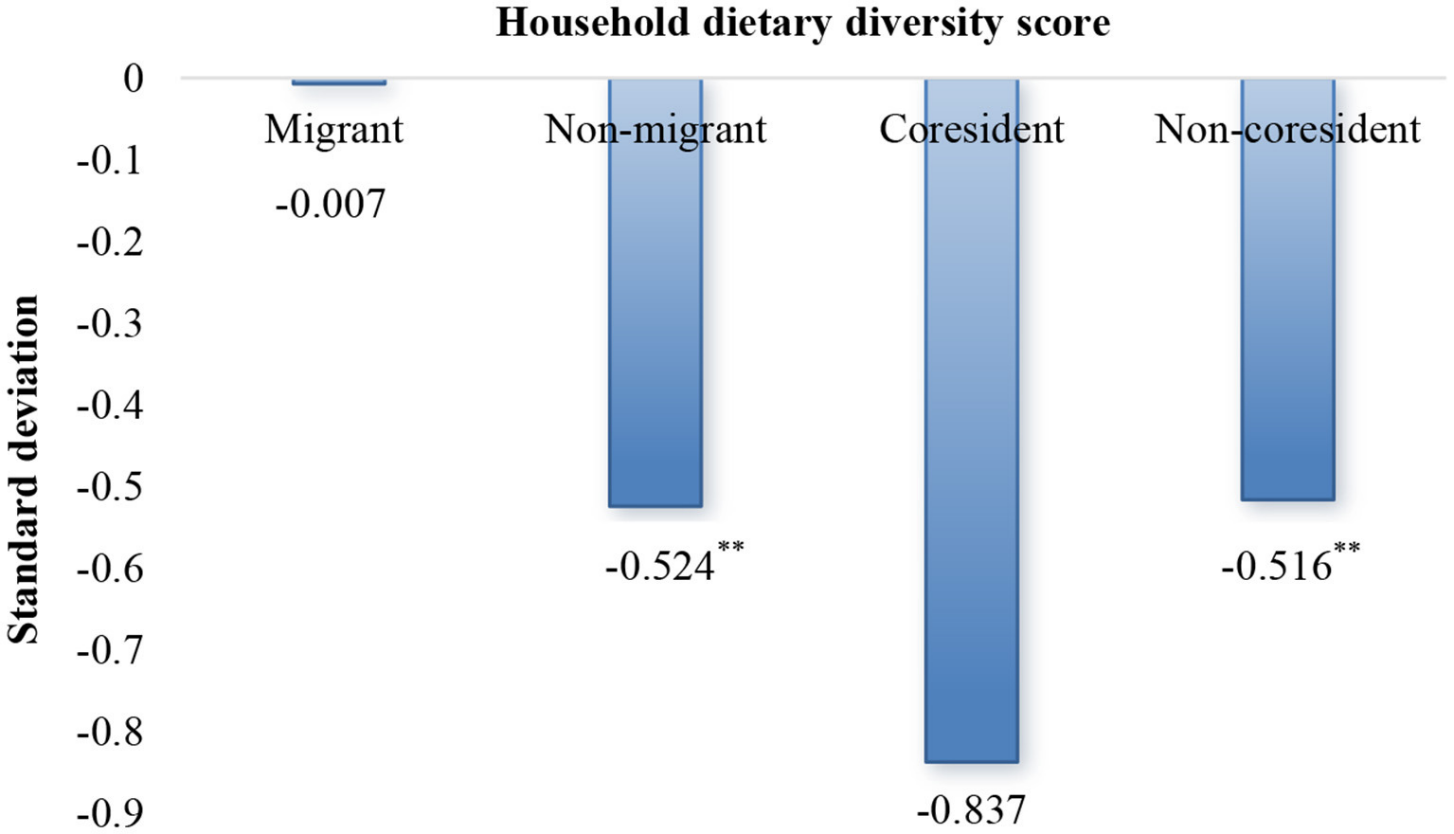
Analytical graph of grouped regression results. ^**^p < 0.05.

Overall, intrahousehold empowerment gaps were found to be detrimental to diet diversity, but differences in dietary quality among different types of farmers deserve further exploration. As shown in Figure 3, the relative empowerment score coefficient of the group comprising non-migrant husband was negative and significant at a 5% level of significance (β = −0.524), and improving women's empowerment level could improve the dietary quality of these farmers. Similarly, gender gaps in the group of non-coresident mothers-in-law had a negative effect on household food security (β = −0.516); narrowing the empowerment gap made it easier to achieve household dietary diversity. For other farmers, there was no correlation between intrahousehold empowerment gaps and dietary diversity.

### 3.4 Robustness test

**Figure 4:**
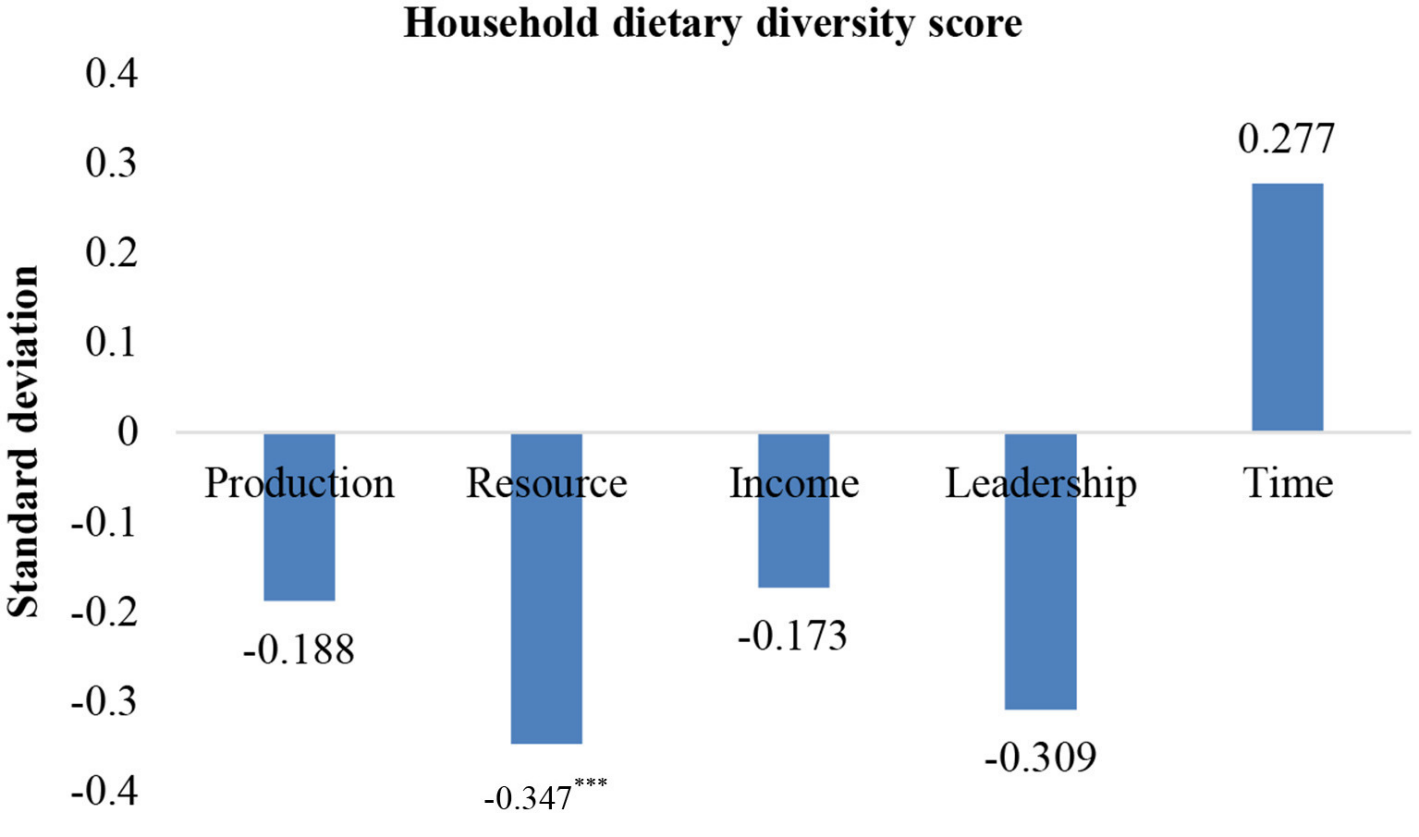
Robustness test of gender gaps and diet quality in different dimensions. Due to spatial limitations, only the regression results of the key explanatory variable are listed; ***p < 0.01.

The results in Table 6 and Figure 4 are consistent with the findings in Tables 4, 5. The relative empowerment score negatively and significantly affected household diet diversity (HDDS9), and the empowerment gaps in the resource domain were negative and significantly correlated with household diet diversity (β = −0.347). The results of the econometric analysis were robust across the different measures of diet diversity.

**Table 6:** Robustness test on the empowerment gaps and dietary diversity.

	**Column 1**	**Column 2**	**Column 3**	**Column 4**
Relative empowerment score	−0.357^*^ (0.191)	−0.338 (0.210)	−0.461^**^ (0.204)	−0.523^***^ (0.194)
Age difference		0.007 (0.010)	0.007 (0.010)	0.006 (0.009)
Education difference		−0.024 (0.019)	−0.035^*^ (0.019)	−0.026^***^ (0.020)
Age of household head			−0.005 (0.006)	−0.001 (0.007)
Education of household head			0.054^**^ (0.023)	0.042^*^ (0.022)
Health status of household head			−0.495^***^ (0.131)	−0.363^***^ (0.134)
Household size				−0.023 (0.051)
Household wealth				0.227^**^ (0.104)
Area of arable land				0.007^**^ (0.004)
Communication expenditure				0.090^**^ (0.046)
Household income				0.006 (0.080)
Social network				0.136^**^ (0.053)
Constant	4.830^***^ (0.062)	4.875^***^ (0.074)	4.978^***^ (0.419)	4.313^***^ (0.914)
*N*	468	468	468	468

^*^p < 0.1, ^**^p < 0.05, ^***^p < 0.01.

### 3.5 Endogeneity test

The regression results for the instrumental variable are shown in Table 7. The first-stage regression results showing that the instrumental variable was significantly positively correlated with the endogenous variable of relative empowerment score (β = 0.181, *P* < 0.001). Furthermore, the minimum eigenvalue statistic value was >10 (12.40), rejecting the null hypothesis of the weak instrumental variable. Compared with the OLS model, the IV-2SLS estimation method did not change the direction of the estimation coefficient of the relative empowerment score of the endogenous variable (β = −3.846). It was statistically significant at a 5% level of significance. Regarding the coefficients of endogenous variables in the two regressions, the IV-2SLS method alleviates the estimation bias caused by OLS endogeneity. Thus, through using the instrumental variable approach, we can also conclude that gender gaps are not conducive to household dietary diversity.

**Table 7:** Results regarding the instrumental variable.

	**OLS**	**IV-2SLS**
Relative empowerment score	−0.563^***^ (0.216)	−3.864^**^ (1.526)
Age difference	0.003 (0.010)	0.008 (0.013)
Education difference	−0.035 (0.022)	−0.037 (0.028)
Age of household head	−0.001 (0.007)	0.010 (0.011)
Education of household head	0.045^*^ (0.025)	0.070^**^ (0.033)
Health status of household head	−0.307^**^ (0.149)	−0.538^**^ (0.216)
Household size	−0.061 (0.060)	−0.035 (0.073)
Household wealth	0.248^**^ (0.116)	0.384^**^ (0.152)
Area of arable land	0.010^**^ (0.005)	0.018^**^ (0.007)
Communication expenditure	0.110^**^ (0.051)	0.094 (0.065)
Household income	0.044 (0.089)	−0.005 (0.114)
Social network	0.113^*^ (0.059)	0.149^**^ (0.076)
Constant	6.118^***^ (1.016)	5.771^***^ (1.297)
Minimum eigenvalue statistic	12.40	
*N*	468	

^*^p < 0.1, ^**^p < 0.05, ^***^p < 0.01.

## 4 Discussion

Our study areas were selected based on the fact that low dietary diversity and inadequate women's empowerment remain significant problems for smallholder farmers in the aforementioned regions. Our findings support the negative association between intrahousehold empowerment score and diet diversity. This study's results are inconsistent with those of Quisumbing et al., who noted that the relative empowerment score has no effect on household diet diversity in some African and Asian countries (34). The results of this study also differ those of from Malapit et al., who reported that relative bargaining power in the household appears to be only weakly correlated with children's nutritional status (25). Therefore, the findings of this paper strengthen the view that promoting gender equality is also brilliant from an economic perspective, increasing productivity and improving other development outcomes, including the prospects for the next generation (21).

The integrative effects of different empowerment domains on household diet diversity are not yet conclusive. Our estimations show that increasing female participation in social/economic groups can help diversify household diets. Similar evidence was found by Malapit, who reported that the gender gaps in group membership are negative and significantly correlated with children's weight-for-age in Bangladesh (18). Our results show that reducing the empowerment gaps pertaining to resource access can improve household diet quality. This is in line with earlier research (10, 12, 32), who found that improving women's status in terms of resources increases the likelihood of more resources being allocated for food consumption and improves diet diversity. In addition, our findings suggest that increasing women's control over income improves dietary diversity in the same households. This finding aligns with earlier studies that show that enhancing women's income decision-making power increases a household's ability to mitigate poverty and ensure food safety (7, 16).

However, the tradeoffs between empowerment domains that we have uncovered are consistent with the findings of the systematic reviews (4, 8, 9) that imply that not all empowerment domains are correlated with food and nutrition security. Our findings advise against assuming that policy measures to address food insecurity and malnutrition will inherently enhance diet diversity outcomes. This caveat parallels Quisumbing et al.'s argument, emphasizing the need to involve women in nutrition-sensitive programs while intervening to protect and enhance their social status, decision-making ability, overall empowerment, and capacity to manage their time, resources, and assets (34). This highlights the essential nature of policies and interventions to empower women through increased decision-making power over assets, enhanced access to credit resources, and improved leadership in their communities.

Expanding social networks in rural areas is a promising strategy to enhance dietary security and nutrition. The coefficient of the variable for social networks is significantly positive, suggesting that social resources contribute to the achievement of dietary diversity. The coefficient of the variable for social networks is significantly positive, suggesting that social resources contribute to the achievement of dietary diversity. Considering evidence from China, two studies found that improving small farmers' levels of social interaction is more effective for alleviating financial hardship and improving nutrition status, mainly because increasing community engagement could bring some material support, as well as the transmission of nutrition and health knowledge and information, among other things (37, 61).

The findings of this study stress the effects of intrahousehold empowerment gaps in reducing food and nutrition insecurity, as well as the heterogeneity among the different groups studied. In particular, gender gaps did not impact dietary diversity in households of husband migrants and residents with mothers-in-law. This is mainly due to the “men migrants, women left behind” strategy, which is adopted to maximize households' benefits (62) and enables the “left-behind women” to be “passively empowered” in the family and agricultural sector. As a result, compared with the relative empowerment score, the impact of women's empowerment score on household dietary diversity was more prominent. The results corroborate a previous study on women's empowerment, wherein it was found that increasing the protein intake of male migrant families by 12.2% was achieved through female empowerment in China (37). Moreover, the mother-in-law is the housekeeper in the traditional Chinese-style rural family (for those co-residing with mothers-in-law), even if the son forms a new family. Therefore, increasing the empowerment levels of daughters-in-law has little effect on improving the family's diet quality.

## 5 Conclusion

This study provides empirical evidence regarding the relationship between the empowerment gap between males and females in the same household and dietary diversity. We confirmed that intrahousehold empowerment gaps are negatively linked to dietary diversity and even more heterogeneity amidst different groups. Overall, increasing female participation in social/economic groups or closing the empowerment gaps regarding access to resources can diversify household diets, while we uncovered that not all empowerment domains are correlated with food security.

Given the prominent problems of food insecurity and malnutrition in rural China, these results provide further justification for policies and interventions that aim to improve women's bargaining position in the household. Thus, this paper serves as an essential reference for promoting changes in food insecurity, malnutrition, and gender dynamics among smallholder households with the implementation of China's rural revitalization strategy. At the same time, policymakers and program designers must be aware of unintended consequences, such as increased group participation, which may increase women's workloads. This means that efforts to achieve diet diversity must be accompanied by measures aimed at changing gender norms and reducing intrahousehold inequality.

However, we also need to recognize the limitations of this study adequately. For one, household dietary diversity is measured based on consumption in the past 24-h recall rather than a long-term retrospective. This may lead to bias in the measurement of food consumption by neglecting the consumption of some main foods (such as fish, meat, etc.); on the other hand, food sources and dietary quality of rural households vary throughout the year, but this paper does not consider the seasonal factors when analyzing food consumption specifically.

## Data availability statement

The datasets used and/or analysed during the current study available from the corresponding author on reasonable request. Requests to access the datasets should be directed to huangyanfang01@caas.cn.

## Author contributions

YH: Data curation, Formal analysis, Investigation, Methodology, Software, Writing—original draft, Writing- review & editing. YY: Writing—original draft, Writing-review & editing. FN: Conceptualization, Funding acquisition, Resources, Supervision, Writing—review & editing. XJ: Conceptualization, Funding acquisition, Resources, Supervision, Writing—review & editing.

## Footnotes

^1^The authors calculated based on survey data.
